# The Role of Bone Scintigraphy with SPECT/CT in the Characterization and Early Diagnosis of Stage 0 Charcot Neuroarthropathy

**DOI:** 10.3390/jcm9124123

**Published:** 2020-12-21

**Authors:** Raju Ahluwalia, Ahmad Bilal, Nina Petrova, Krishna Boddhu, Chris Manu, Prashanth Vas, Maureen Bates, Ben Corcoran, Ines Reichert, Nicola Mulholland, Venu Kavarthapu, Gill Vivian, Michael Edmonds

**Affiliations:** 1 Department of Orthopedics, King’s College Hospital, London SE5 9RS, UK; abilal@nhs.net (A.B.); k.boddu@nhs.net (K.B.); ines.reichert@kcl.ac.uk (I.R.); venu.kavarthapu@nhs.net (V.K.); 2 Diabetic Foot Clinic, King’s College Hospital, London SE5 9RS, UK; nina.petrova@nhs.net (N.P.); chris.manu@nhs.net (C.M.); prashanth.vas@nhs.net (P.V.); mbates2@nhs.net (M.B.); Michael.edmonds@nhs.net (M.E.); 3 Department of Diabetes, Faculty of Life Sciences and Medicine, King’s College, London SE5 9RS, UK; 4 Department of Nuclear Medicine, King’s College Hospital, London SE5 9RS, UK; bcorcorn@nhs.net (B.C.); nicolamulholland@nhs.net (N.M.); gillvivian@gmail.com (G.V.)

**Keywords:** SPECT/ CT, Charcot neuroarthropathy, Stage 0 Charcot neuroarthropathy, diabetes, fracture

## Abstract

We describe the use of Single Photon Emission Computed Tomography/Computed Tomography (SPECT/CT) in the investigation and diagnosis of Charcot neuroarthropathy (CN) in patients with a hot swollen foot but normal radiographs and clinical suspicion of CN, usually termed Stage 0. This was a retrospective cohort review of 46 diabetes patients who underwent 3 phase bone scintigraphy with “High Resolution” SPECT/CT. The imaging demonstrated that Stage 0 Charcot foot has a distinct bone pathology, which can be classified into three groups: (1) fractures on Computed Tomography (CT) with accompanying focal uptake of tracer on SPECT, (2) bony abnormalities apart from fracture on CT with focal uptake of tracer on SPECT, and (3) normal CT but focal bony uptake of tracer on SPECT. The CT component of SPECT/CT detected bony fractures in 59% of patients. Early treatment with below knee cast and follow-up for 24 months showed only 4 patients who developed Stage 1 Eichenholtz Charcot foot. Our findings support the use of 3 phase bone scintigraphy with SPECT/CT in the characterization and early diagnosis of CN. Stage 0 Charcot foot has a distinct bone pathology which requires urgent treatment to prevent progression to Stage 1 Eichenholtz Charcot foot. If SPECT/CT is unavailable, CT alone will detect bone fracture in 59% patients.

## 1. Introduction

Charcot neuroarthropathy (CN) is a condition affecting the bones, joints, and soft tissues of the foot and ankle, characterized by inflammation in the earliest phase [1]. Pathogenesis is not fully understood, although, recently, autoantibodies against oxidative post-translational modified collagen, particularly type 2 collagen have been noted in CN and diabetic neuropathy, suggesting the possible involvement of autoimmunity [2]. In its severest form CN can lead to deformity, ulceration, infection, and amputation. It contributes to significant morbidity and premature mortality and has a negative impact on the activities of daily living [1]. Although procrastination and non-treatment will lead to deformities and ulceration [3], initiation of off-loading at an early stage may prevent progression of disease and reduce the incidence of deformity [4].

A characteristic early presentation in diabetes is the hot swollen foot often when radiographs are still normal. This is the recognized Stage 0 Charcot foot [5,6,7]. However, the pathology at this stage is not well understood although Magnetic Resonance Imaging (MRI) has proved helpful by identifying reactive, inflammatory bone marrow with or without microfracture before overt radiographic changes are apparent [4,8]. The previous use of three-phase bone scintigraphy in diabetes patients with neuropathy suggested increased blood flow to bone but the planar imaging has low anatomical resolution [9]. However, the introduction of hybrid Single Photon Emission Computed Tomography/ Computed Tomography (SPECT/CT) in nuclear medicine has improved the diagnostic potential of bone scintigraphy [10]. Combining SPECT and CT considerably increases bone scan image quality (attenuation correction), anatomic localization and diagnostic accuracy [11].

We have used SPECT/CT as a functional and structural imaging modality to identify initial blood flow and bone abnormalities in diabetes patients presenting with a hot swollen foot who thus have a suspected diagnosis of CN. The aim of the study was to assess if there were characteristic changes on the SPECT-CT of the unilateral hot swollen foot which is clinically in the early stages of CN.

## 2. Methods

### 2.1. Patients

The inclusion criteria consisted of people with diabetes (Type 1 or Type 2), who presented to the Multidisciplinary Diabetic Foot Clinic between 2010 and 2013 with an acute (active) hot swollen foot but with intact skin and with radiographically normal bones and joints. Skin temperature was to be >2 °C compared with the same site on the contralateral foot (Dermatemp 1000; Exergen, Watertown MA, USA).

Exclusion criteria included all patients who had a previous history of CN in either foot or associated deformity in either foot, a history of amputation or surgery in either foot, and any signs or symptoms of ulceration.

Below-knee casting was initiated at the time of clinical presentation. Each patient underwent a SPECT/ CT scan within 2 weeks of clinical presentation in line with our routine method of clinical investigation. The cast was removed immediately before the bone scan and then replaced after the bone scan had been completed on the same day. All patients were treated as per protocol for an acutely diagnosed Charcot process with off-loading comprising a below knee cast until the clinical presentation had normalized.

### 2.2. Single Photon Emission Computed Tomography/Computed Tomography (SPECT/CT) Scan

**Figure 1:**
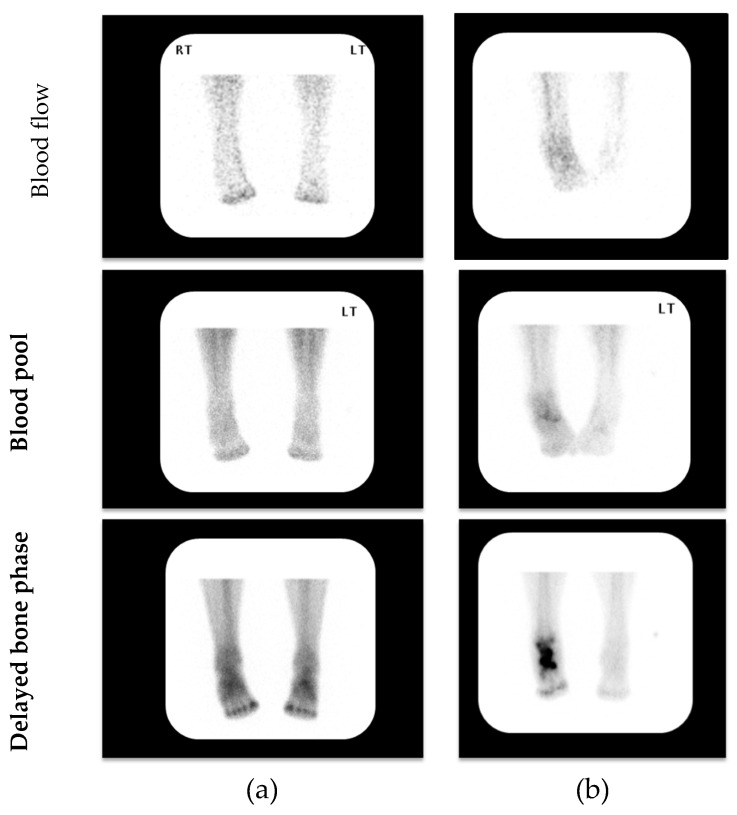
Representative examples of 3-phase bone scintigraphy assessing blood flow, blood pool and delayed bone phases in patients with clinically suspected acute (active) Charcot neuroarthropathy (CN); (**a**) 3-phase negative scan; (**b**) 3-phase positive scan. (LT—Left) (RT—Right)

All patients were imaged in the Department of Nuclear Medicine at King’s College Hospital, London. Patients were placed supine with feet supported in neutral rotation facing the imaging camera (Siemens^®^ Symbia True Point 16 SPECT/ CT gamma camera; Siemens AG Siemens Avanto 1.5T, Erlangen, Germany) with a single bed position to include both feet and ankles during all phases. Patients were injected with a gamma emitting radiopharmaceutical 800MBq Tc-99m MDP (technetium-99m methylene diphosphonate). The specific protocol consisted of a Triple Phase Bone Scan comprising blood flow, blood pool and delayed bone phases (Figure 1). Imaging with SPECT allowed the capture of 3D images with detection heads that rotate 360 degrees. Finally, a CT scan was acquired and assessed using fine cut “High Resolution” 1 mm images. Simultaneously, SPECT/CT fusion images were reconstructed using the Siemens^®^ Flash 3D reconstruction that incorporates attenuation correction and resolution recovery.

### 2.3. Assessment of Single Photon Emission Computed Tomography/Computed Tomography (SPECT/CT) Imaging & Outcome Analysis

A working group consisting of Nuclear Medicine Physician, Orthopedic Surgeon, and Diabetologist conducted a high-level retrospective review of all imaging (G.V., R.A., and M.E.) taken at the time of the index diagnosis of the newly hot swollen foot. All scans were assessed using GE Centricity PACS (GE Healthcare, Barrington, IL 60010, USA) workstations with dedicated high-resolution viewing monitors and fused images on the Hermes Medical Solutions Hybrid Viewer (Stockholm, Sweden). All scans were blinded and retrospectively reviewed in a sequential coordinated manner using an algorithmic approach. The main outcome of the study was the characterization of the individual components of the SPECT-CT in a group of patients that had a clinical diagnosis of a unilateral CN, the primary outcome measure being the constituent differences between the parts of the scan on the affected foot compared to the non-affected contralateral foot.

In the final analysis, the scans were divided into 3 discrete groups depending on the findings of the CT component, namely Group 1 showing the presence of fractures, Group 2 the presence of bony abnormalities but no fracture, and Group 3 no fracture or bony abnormalities.

### 2.4. Clinical Management

The overall management followed clinic protocol as determined by Clinical Guideline 10 of the National Institute for Clinical Excellence (NICE) (2004) [12] and used the previously reported diagnostic approach uninfluenced by the SPECT CT findings [13]. All subjects were followed up in the Multidisciplinary Diabetic Foot Clinic initially weekly and thereafter every 2–3 weeks for cast review and to monitor resolution of the CN process. Resolution was defined by clinical reduction of swelling and heat as indicated by a foot skin temperature difference of less than 2 °C between the feet at two consecutive clinical visits.

Patients were also monitored to detect the development of bone and joint changes of Stage 1 Eichenholtz Charcot foot on radiographs including subluxation, dislocation, and bony fragmentation, despite optimal immobilization.

All patients were followed for a minimum of 2 years. The study was reviewed by our research and governance team and, as it comprised standard investigation and follow up in the Diabetic Foot Clinic and there was not any additional intervention on the patients, it was deemed not to require ethical approval.

### 2.5. Statistical Analysis

This is primarily an observational study, and demographics, comorbidities, clinical features, diagnostic tests, off-loading treatment, and duration, were expressed in frequency and percentages.

## 3. Results 

### 3.1. Patients & Demographics

Forty-six patients with diabetes and normal radiographs of both feet but with a high clinical suspicion of CN in the unilateral hot swollen foot were investigated. Their mean age at the time of presentation was 57 years (range 34–76 years), mean HbA_1_C (Glycated hemoglobin) was 8.4% (range 6.8–14.7%)/68 mmol/mol (range 51–137 mmol/mol), and there were 32 males and 24 females.

### 3.2. Results of Bone Scans with Single Photon Emission Computed Tomography/Computed Tomography (SPECT/CT)

The results of the bone scintigraphy with SPECT/CT scans were classified into three groups and individual details pertaining specifically to the unilateral hot swollen foot are presented in Table 1, Table 2, and Table 3.

#### 3.2.1. Group 1: Patients with Fracture(s) on CT and Focal Uptake of Tracer on Single Photon Emission Computed Tomography (SPECT)/) (Table 1)

In group 1, there were 27 patients, in whom the foot showed unilateral increased blood flow, blood pool, and tracer uptake in the delayed phases with multiple focal areas of tracer uptake on the SPECT (Table 1). These feet also had bony abnormalities on the CT scan that were not seen on the original radiographs, consisting of definitive fractures, cortical breaches (which were considered to be uni-cortical fractures), bony fragmentation, cysts, and lucencies.

There were 25 definitive fractures being either un-displaced or minimally displaced, including 4 calcaneal fractures, 3 talar fractures, 3 fibula fractures and 4 mid-foot fractures (involving the navicular, medial, middle, and lateral cuneiforms, cuboid), and 11 forefoot fractures of metatarsals and phalanges. We also observed 11 uni-cortical fractures. Five feet showed bone fragmentation. Nine feet showed other bony lesions, as well as fractures, and these comprised cysts and lucencies. Two feet were observed to have co-existing degenerative changes in the 1st metatarsal-phalangeal joint, one with osteophytes.

**Figure 2:**
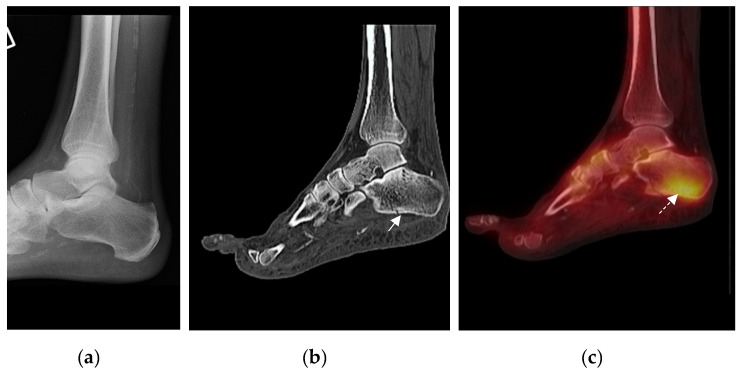
Representative example of imaging assessment of a patient presenting with a unilateral hot swollen foot and clinically suspected acute active Charcot Neuroarthropathy (CN). (**a**) Lateral foot/ ankle radiograph with no bone abnormality (incidental finding of vascular calcification); (**b**) CT shows calcaneal fracture (arrow) not noted on original radiograph; (**c**) increased uptake within the calcaneum (arrow). Uptake is centered around the fracture, showing correlation between Single Photon Emission Computed Tomography (SPECT) and CT (Computed Tomography) on fusion imaging.

In total, 17 feet showed multiple pathology comprising either one or more fractures or fracture accompanied by bony fragmentation or other bony lesions (excluding the degenerative changes at the 1st metatarsal-phalangeal joint), (Table 1). The fusion images showed correlation between the sites of fractures and bony abnormalities and the sites of focal tracer uptake (Figure 2). However, in some patients, the sites of focal tracer uptake on the SPECT were more frequent and widespread than the bony abnormalities.

There were 10 fractures which were considered as possible avulsion fractures as a result of the location of SPECT activity and the concurrent features of fracture and fragmentation on CT. From the site of the increased tracer uptake associated with the avulsion fracture, the possible ligament or tendon responsible for the avulsion was inferred and included the Lisfranc (medial cuneiform and base of 2nd metatarsal) and Spring ligaments (navicular and cuneiforms) and the Extensor Hallucis Longus (proximal phalanx), Tibialis Anterior (medial and middle cuneiform), and Achilles tendons (calcaneum).

The features of increased blood flow and blood pool accompanied by focal tracer uptake on the SPECT, and the fractures and other bony abnormalities on the CT in Table 1 were interpreted as representing the early features of CN, and the patients were treated with a below knee cast.

#### 3.2.2. Group 2: Patients with Bony Abnormalities Apart from Fracture on CT and Focal Uptake of Tracer on SPECT (Table 2)

Group 2 consisted of 9 patients with bony abnormalities on the CT scan (but no fractures), 8 of whom had increased unilateral blood flow and blood pool and increased tracer uptake in the delayed phase with focal areas of uptake on the SPECT in the suspected foot (Table 2). One patient had an equivocal blood pool phase, but the blood flow was increased. Three patients had erosions, 4 patients had subarticular cysts and 2 patients showed areas of bony lucency. The SPECT showed focal areas of uptake corresponding to these bony lesions apart from patient 2, in whom the navicular cyst was not associated with focal tracer uptake, but there was focal uptake at the base of the 5th metatarsal. As well as showing focal areas of tracer uptake corresponding to all other bony lesions, the SPECT demonstrated focal areas of tracer uptake which appeared normal on CT scan. In addition, one patient showed degenerative change in the 1st metatarsal-phalangeal joint on CT.

The observations combining increased blood flow and blood pool with bony abnormalities associated with focal uptake of tracer on SPECT were interpreted as representing the early features of CN, and the patients were also treated with a below knee cast.

#### 3.2.3. Group 3: Patients with Normal CT Findings But Focal Uptake of Tracer on SPECT (Table 3)

This group consisted of 10 patients having increased unilateral blood flow and blood pool with focal areas of uptake on the SPECT, but the CT was normal. Seven feet had multiple sites of focal uptake on the SPECT. These may have represented the earliest abnormalities of bone turnover with increased osteoblastic activity in response to a possible minor injury and thus constituted a very early Charcot foot, and these patients were treated with a below knee cast.

One patient had focal tracer uptake in the navicular, medial, and middle cuneiforms with increased blood flow and blood pool imaging compatible with early CN together with focal uptake at the proximal site of the attachment of the plantar fascia in the calcaneum suggestive of plantar fasciitis. Two patients had increased uptake at the insertion of the Achilles tendon, and one of them also had focal uptake of tracer at the proximal attachment of the plantar fascia to the calcaneum indicative of plantar fasciitis [14]. 

## 4. Clinical Outcome

Four patients proceeded to bone and joint disruption characteristic of Stage 1 Eichenholtz Charcot foot, even with appropriate standard management. These developed within three months of initial presentation, whilst the patients were still under standard treatment with a below knee cast for CN. Three of the patients, who progressed to Stage 1 Eichenholtz Charcot foot, were considered at presentation to have increased blood flow and blood pool and focal uptake on the SPECT and discrete fracture on the CT. However, one patient, despite having increased blood flow and blood pool and focal uptake on the SPECT, as well as a normal CT, still developed bone and joint disruption whilst in the cast consistent with Eichenholtz Stage 1. It was important to note that the site of bony disruption of the foot in Eichenholtz Stage 1, in all four patients, correlated with the sites of focal uptake of tracer on the SPECT. These patients eventually went on to Stage 2 and 3 Eichenholtz Charcot foot with consolidation but retained a plantigrade foot with cast treatment.

## 5. Discussion

In this report, SPECT/CT has demonstrated that there is distinct bone pathology, predominantly fracture, in the hot swollen foot presenting as Stage 0 Charcot foot. There has been considerable interest in characterizing this stage both to facilitate early diagnosis and also to understand the pathogenesis of this condition. MRI is considered the main mode of investigation at this stage (as it is in our institution), and Chantelau et al. have highlighted closed subcortical trabecular microfractures (bone bruise) as an early diagnostic sign [4]. The present report classifies functional and structural abnormalities in Stage 0 as observed on 3 phase bone scintigraphy with SPECT/CT into three groups as noted in Table 1, Table 2, and Table 3.

The most frequent bony abnormality, as described in group 1, was fracture on CT, which was associated with unilateral increased blood flow and blood pool and focal tracer uptake on the SPECT, indicating increased vascularity and osteoblastic response. The unilateral increase in blood flow and blood pool was most probably secondary to fracture, although the concomitant neuropathy associated with CN may have also contributed. Fractures have been well described as radiological features of Stage 1 Charcot foot [15], but the SPECT/CT has highlighted a spectrum of bony abnormalities, including fractures at Stage 0. Definitive fractures were observed, as well as cortical breaches or uni-cortical fractures. Although single bone fractures were observed in some feet, multiple fractures were noted in others, and these have previously been reported in CN in diabetes and other neuropathic conditions, including congenital insensitivity to pain [16,17]. Although a positive bone scan has been reported in Stage 0 [6], the addition of CT has emphasized the presence of fractures in Stage 0.

Avulsion fractures were recognized in the spectrum of fractures and were assumed to have been induced by traction from the Lisfranc and Spring ligaments and from the Achilles, Extensor Hallucis Longis, and Tibialis Anterior tendons. Focal tracer uptake conformed to the expected subchondral location of these ligament and tendon insertions and this may reflect abnormal bone turnover at these sites. Calcaneal posterior tuberosity avulsion fractures have been noted previously in diabetes [18]. The concept of calcaneal avulsion fractures and their association with CN was first introduced in 1991 by Kathol et al. [19]. Since then, these fractures have been further classified into Sanders/ Frykberg pattern V or Brodsky type 3b Charcot foot. It is possible that avulsion fractures may be a fundamental event in the early natural history of CN [20].

Erosions, cysts, and lucencies were also observed in patients presenting with a hot swollen foot and were usually associated with focal uptake of tracer on the SPECT and increased blood flow and blood pool phases. Ultrasound studies of patients in Stage 0 Charcot foot with normal radiography have recently reported the presence of juxta-articular erosions [21]. MRI has demonstrated subchondral cysts in the metatarsal/ tarsal and tarsal regions in Stage 0 [22]. Furthermore, in a study of the natural history of bone marrow oedema as shown on the MRI, subchondral cysts have been observed to evolve from bone marrow oedema [23]. Areas of bone lucency probably reflect inflammatory osteolysis, which has been reported as a feature of acute (active) CN. Furthermore, excessive osteoclastic activity in the environment of cytokine mediators of bone resorption interleukin-1 (IL-1), IL-6, and tumor necrosis factor alpha (TNF-alpha) has been reported [24]. Serum inflammatory markers are also raised in acute CN and can enhance bone resorption through the stimulation of osteoclastic progenitor cells, as well as mature osteoclasts [25,26].

In the third group, patients had normal CT imaging but increased focal uptake of tracer in the SPECT, as well as increased blood flow and blood pool. The patients fell into 2 groups, those with probable early CN and those with miscellaneous conditions. In the first group, the focal activity on the SPECT revealed a functional abnormality of increased osteoblastic activity, which may be the primary response to a trauma and the first event in CN. Indeed, one patient in this group developed Stage 1 CN despite casting treatment. However, the presence of focal tracer uptake on the SPECT in the hot swollen foot despite a normal CT raises the opportunity of diagnosing the Charcot foot at Stage 0 before CT changes develop. The second group of patients showed increased tracer uptake at the sites of Achilles ligament insertion and also at the attachment of plantar fascia, indicating probable diagnoses of Achilles tendonitis and plantar fasciitis. This demonstrated the usefulness of SPECT/CT in identifying these conditions, which could be treated with standard therapies. Although Achilles tendonitis is a separate diagnosis from CN, it is interesting to note that a recent ultrasound study reported tenosynovitis as a feature of CN [21].

## 6. Limitations

This is the first comprehensive report on characterizing the changes of early CN on SPECT/ CT. However, it is retrospective and biased by being based on routine clinical material. Even so, the study population was followed-up until “healing of that episode”. It is important to note that the CT component of the SPECT/CT detected bony fracture in 59% of cases and a bone abnormality including fractures in 78% of our patients presenting with a hot swollen foot, and it might be argued that CT alone, which is more readily available than SPECT/CT or MRI, could be the initial investigation to detect bone pathology in the suspected Charcot foot. At present, MRI remains the main method of investigation of Stage 0, although recently ultrasound and 18F-FDG PET/CT scanning (18-fluorodeoxyglucose positron emission tomography/computed tomography) have been used [7,21]. But we did not specifically test 3 phase bone scintigraphy with SPECT/CT against these modalities. SPECT/CT may have some advantages over MRI in identifying fractures and cysts, especially in those patients where MRI is contraindicated [27]. Further studies of this modality will be required to assess the validity and demonstrate the inter- and intra-observer agreement between investigators.

## 7. Conclusions

Delay in the diagnosis of Stage 0 Charcot foot can result in severe disruption of the bony architecture [28]. SPECT/CT is a useful functional and structural imaging modality in the evaluation of patients suspected of having a Stage 0 Charcot foot. It has identified increased blood flow and bony changes that may be the initial events in Charcot pathophysiology and also aids the early diagnosis of CN in patients presenting with a hot swollen foot. SPECT/CT demonstrates that, even in Stage 0 Charcot foot, there is distinct bone pathology, predominantly fracture, which demands appropriate urgent treatment. If SPECT/CT is not easily available, then CT alone could be carried out as it may identify 59% of the bony lesions.

## Figures and Tables

**Table 1:** Group 1: Patients with increased blood flow and blood pool and areas of focal uptake of tracer on Single Photon Emission Computed Tomography (SPECT) with fracture(s) on Computed Tomography (CT). A positive (+ve) result indicates increased tracer uptake in the phase of the bone scan as designated in the Table heading.

Patient Number	Blood Flow	Blood Pool	Delayed Phase	SPECT (Areas of Increased Uptake)	CT
1	+ve	+ve	+ve	Medial and middle cuneiforms and navicular and proximal phalanx 1st ray	Fracture proximal phalanx and fracture 1st metatarsal head
2	+ve	+ve	+ve	Medial forefoot, mid-foot, dome of the medial talus and 1st metatarsal head	Fracture talus and a uni-cortical fracture 1st metatarsal head
3	+ve	+ve	+ve	Head of talus and body of navicular, (talo-navicular joint) and lateral cuneiform, 4th and 5th metatarsal bases	Fracture (avulsion) distal lateral cuneiform, and fracture base of 5th metatarsal
4	−ve	+ve	+ve	Head of 1st metatarsal 5th metatarsal base	Lucency 1st metatarsal head and fracture base of 5th metatarsal
5	+ve	+ve	+ve	Heads of 1st, 2nd and 3rd metatarsals (1st, 2nd and 3rd metatarsal-phalangeal joints)	Fracture head of 1st metatarsal with subarticular lucency and uni-cortical fractures 2nd and 3rd metatarsal heads and bases
6	+ve	+ve	+ve	Neck and body of talus	Fracture (avulsion) talar neck and subarticular fracture of posterior talar dome
7	+ve	+ve	+ve	Dome of talus, sustentaculum tali, navicular, fibula, and medial cuneiform	Fracture navicular and cyst fracture, multiple subarticular cysts of navicular
8	+ve	+ve	+ve	Calcaneum and cuboid, (calcaneo-cuboid joint), navicular, medial cuneiform, Base of 1st metatarsal (tarso-metatarsal joint), and base of 2nd metatarsal	Uni-cortical fracture (avulsion) navicular, and subarticular cyst of navicular
9	+ve	+ve	+ve	Inferior and medial aspect of medial cuneiform	Uni-cortical fracture (avulsion) medial cuneiform, subarticular cysts of navicular
10	+ve	+ve	+ve	Head and base of 1st metatarsal	Fractures head and base of 1st metatarsal
11	+ve	+ve	+ve	Medial cuneiform	Fracture 3rd toe and subarticular cyst medial cuneiform
12	+ve	+ve	+ve	Distal and middle phalanx of 1st toe	Uni-cortical fracture (avulsion) dorsal proximal phalanx and fragmentation
13	+ve	+ve	+ve	Navicular and medial cuneiform	Uni-cortical fracture (avulsion) navicular and periarticular cysts navicular
14	+ve	+ve	+ve	Left 5th toe	Fracture proximal third of 5th toe
15	+ve	+ve	+ve	Calcaneal body	Fracture calcaneum
16	+ve	+ve	+ve	Medial cuneiform	Fracture (avulsion) medial cuneiform, degenerative change 1st metatarsal-phalangeal joint
17	+ve	+ve	+ve	Fibula, posterior talus and distal tibia (tibio-talar joint)	Fracture fibula and associated cortical fragmentation of distal third fibula
18	+ve	+ve	+ve	Osteophyte from medial cuneiform to middle cuneiform	Fracture of cuneiform bridging osteophyte
19	+ve	+ve	+ve	Proximal 4th phalanx, 4th and 5th metatarsals and dorsal talar neck	Fracture proximal third 4th phalanx
20	+ve	+ve	+ve	Posterior third of calcaneum	Fracture posterior calcaneum
21	−ve	+ve	+ve	Base of 5th metatarsal	Uni-cortical fracture base 5th metatarsal Cystic change in lateral cuneiform, irregularity talo-calcaneal joint, and anterior talar lip fragmentation
22	+ve	+ve	+ve	Cuboid and posterior talus	Fracture superior articular surface of cuboid, degenerative change 1st metatarsal head and osteophytes
23	+ve	+ve	+ve	Distal calcaneum, posterior talus and fibula	Fragmentation of cuboid, and fracture (avulsion) distal third fibula
24	+ve	+ve	+ve	Anterior facet of calcaneum and proximal cuboid (calcaneo-cuboid joint)	Left calcaneal anterior process fracture
25	+ve	+ve	+ve	Navicular and middle cuneiform	Uni-cortical fracture and fragmentation of the middle cuneiform
26	+ve	+ve	+ve	Lateral malleolus (Fibula)	Fracture (avulsion) distal fibula
27	+ve	+ve	+ve	Posterior calcaneum at the insertion of Achilles tendon	Fracture (avulsion) of posterior third of calcaneum

**Table 2:** Group 2. Patients with increased blood flow and blood pool and areas of focal uptake of tracer on Single Photon Emission Computed Tomography (SPECT) and bony abnormalities on Computed Tomography (CT). The lesions noted on CT were associated with focal increased uptake of tracer on the SPECT except for Patient 2, in whom the navicular cyst was not associated with focal tracer uptake, but there was focal uptake of tracer at the base of the 5th metatarsal. A positive (+ve) result indicates increased tracer uptake in the phase of the bone scan as designated in the Table heading.

Patient Number	Blood Flow	Blood Pool	Delayed Phase	SPECT (Areas of Increased Uptake)	CT
1	+ve	+ve	+ve	1st, 2nd and 3rd Metatarsal bases Medial, middle and lateral cuneiforms, (1st, 2nd and 3rd tarso-metatarsal joints), talus, and calcaneum	Erosions medial cuneiform
2	+ve	+ve	+ve	Base of 5th metatarsal	Navicular cyst
3	−ve	+ve	+ve	Medial cuneiform, 2nd and 3rd metatarsal bases	Cyst middle cuneiform and 2nd metatarsal and subarticular cystic lesions medial cuneiform
4	+ve	+ve	+ve	1st Metatarsal head	Lucency 1st metatarsal head
5	+ve	−ve	+ve	1st Metatarsal head	Cyst 1st metatarsal head
6	+ve	+ve	+ve	1st Metatarsal base and medial cuneiform	Erosions 1st metatarsal base and medial cuneiform and Erosions in 1st metatarsal head
7	+ve	−ve	+ve	Medial cuneiform,1st and 2nd metatarsal bases	Erosions medial cuneiform and 1st and 2nd metatarsal bases
8	+ve	+ve	+ve	Base of cuboid and distal anterior calcaneum (calcaneo-cuboid joint)	Subarticular cysts base of cuboid
9	+ve	+ve	+ve	Middle cuneiform and navicular, 3rd metatarsal	Lucency middle cuneiform, degenerative change 1st metatarsal-phalangeal joint

**Table 3:** Group 3: Nine patients had increased blood flow and blood pool phases, and Patient 3 had increased blood pool but not increased blood flow. All patients had areas of focal uptake on Single Photon Emission Computed Tomography (SPECT) but no bony abnormality on Computed Tomography (CT). A positive (+ve) result indicates increased tracer uptake in the phase of the bone scan as designated in the Table heading.

Patient Number	Blood Flow	Blood Pool	Delayed Phase	SPECT (Areas of Increased Uptake)	CT
1	+ve	+ve	+ve	Navicular, calcaneo-talar, tibio-talar, and medial cuneiform	−ve
2	+ve	+ve	+ve	Base of 1st and 2nd metatarsals, medial cuneiform and distal part of middle cuneiform	−ve
3	−ve	+ve	+ve	2nd, 3rd, 4th and 5th metatarsal bases and medial, middle and lateral cuneiforms and cuboid (2nd, 3rd, 4th and 5th tarso-metatarsal joints)	−ve
4	+ve	+ve	+ve	Lateral cuneiform	−ve
5	+ve	+ve	+ve	Superior calcaneum and tibial deltoid insertion	−ve
6	+ve	+ve	−ve	Navicular, medial cuneiform, middle cuneiform and 1st metatarsal base and sesamoids	−ve
7	+ve	+ve	+ve	Bases of 1st, 2nd and 3rd metatarsals and medial, middle and lateral cuneiforms (1st, 2nd and 3rd tarso-metatarsal joints)	−ve
8	+ve	+ve	+ve	Navicular, medial and middle cuneiforms, and insertion of plantar fascia	−ve
9	+ve	+ve	+ve	Insertion of Achilles tendon at calcaneum and 2nd metatarsal head	−ve
10	+ve	+ve	+ve	Insertion of Achilles tendon at calcaneum and insertion of plantar fascia	−ve
